# A prospective, observational study of patients with uncommon distal symmetric painful small-fiber neuropathy

**DOI:** 10.1371/journal.pone.0183948

**Published:** 2017-09-28

**Authors:** Jung-Lung Hsu, Ming-Feng Liao, Hui-Ching Hsu, Yi-Ching Weng, Ai-Lun Lo, Kuo-Hsuan Chang, Hong-Shiu Chang, Hung-Chou Kuo, Chin-Chang Huang, Long-Sun Ro

**Affiliations:** 1 Department of Neurology, Chang Gung Memorial Hospital, Linkou Medical Center and Chang Gung University College of Medicine, Taipei, Taiwan; 2 Taipei Medical University, Graduate Institute of Humanities in Medicine, Taipei, Taiwan; 3 Taipei Medical University Research Center for Brain and Consciousness, Shuang Ho Hospital, New Taipei City, Taiwan; 4 Department of Traditional Chinese Medicine, Division of Chinese Acupuncture and Traumatology, Chang Gung Memorial Hospital, Linkou Medical Center and Chang Gung University College of Medicine, Taipei, Taiwan; Weill Cornell Medical College in Qatar, QATAR

## Abstract

**Objective:**

To investigate the clinical characteristics of patients with uncommon distal symmetric painful small-fiber neuropathy (DSPSFN).

**Methods:**

From September 2012 to September 2014, participants between 18–70 years of age that had DSPSFN defined by clinical signs/symptoms and ID pain > 2 or DN4 > 4 on questionnaires for more than 1 month were included. Participants who had previous historical or laboratory evidence of common etiologies of DSPSFN were excluded. Enzyme activity and genetic studies for Fabry diseaseand familial amyloid polyneuropathy were performed after participants fulfilled the inclusion and exclusion criteria. The cryoglobulin test, autoantibodies studies and electrophysiological studies were performed in these participants.

**Results:**

In total, 100 cases were enrolled in the current study. Three cases of subclinical diabetes mellitus and two cases of fibromyalgia were found. Fabry disease (1%) and familial amyloid polyneuropathy (3%) with Ala97Ser transthyretin (TTR) mutations were also detected. The cryoglobulin test was positive in 30% of participants, and these participants had higher DN4 scores than the negative group. In the autoantibodies studies, 59% of the participants had abnormal anti-Ro/SSA and/or anti-La/SSB antibodies.

**Conclusions:**

Cryoglobulinemia is not a rare etiology of uncommon DSPSFN. The long-term prognosis is quite good in these participants. From our structuralized protocol, Fabry disease and familial amyloid polyneuropathy could be easily detected in these cases of uncommon DSPSFN.

## Introduction

Distal symmetric painful small-fiber neuropathy (DSPSFN) is a clinical condition characterized by chronic, severe neuropathic pain and involves unmyelinated C and thinly myelinated Aδ fibers. The characteristics of pain symptoms include burning, shooting pain, paresthesia or allodynia. The distribution pattern of the pain is in the distal four limbs, especially in the fingers and toes. From routine nerve conduction studies (NCSs), small fibers are undetectable; however, their damage frequently causes a neuropathic pain syndrome, making the diagnosis of small-fiber neuropathy (SFN) often particularly difficult. Correct diagnosis of SFN is based on clinical diagnostic criteria including (i) clinical signs/symptoms of small-fiber impairment or dysfunction (pinprick and thermal sensory loss and/or allodynia and/or hyperalgesia), the distribution of which is consistent with peripheral neuropathy (length or non-length-dependent neuropathy); (ii) abnormal warm and/or cooling threshold at the foot assessed by quantitative sensory testing; and (iii) a nerve biopsy study [1]. The incidence and prevalence of DSPSFN is unknown, but it is probably not a rare disease [2]. For example, diabetes and chronic kidney disease have often been reported to be associated with SFN [3, 4]. A recent study performed in the Netherlands showed that the incidence of SFN was 11.7 cases/100,000 inhabitants/year with a prevalence of 52.95cases/100,000[5]. As the chronic pain and uncomfortable sensation in painful neuropathy may be intolerable to the patient, the impact on the quality of life (QoL) has been studied, demonstrating a severe overall reduction in the QoL in SFN patients[6].

The etiology of DSPSFN is associated with various disorders. It may be associated with systemic diseases such as metabolic disorders (diabetes[3], hypothyroidism[7], chronic kidney disease[4]), infectious disorders (HIV[8], hepatitis C[9]), toxic exposure and substance abuse (alcohol abuse[10], nitrofurantoin[11]), immune-mediated disorders (amyloidosis[12], vasculitis[13], cryoglobulinemia[14]) and hereditary disorders (Fabry disease[15], familial amyloidosis[12]). However, the proportion of instances of the different etiologies is unknown. Despite a comprehensive work-up in patients with SFN, the proportion of individuals diagnosed with idiopathic or cryptogenic forms remains substantial, ranging from 30% to 42% in different data sets[1, 16]. Of all of the etiologies of SFN, cryoglobulinemia-related painful neuropathy may be a rare but potentially treatable disorder[17]. Cryoglobulins are immunoglobulins that precipitate at low temperatures and re-dissolve upon rewarming. The clinical manifestation of cryoglobulinemia includes skin purpura, arthralgia and neuropathy. Currently, there are insufficient epidemiological studies on the prevalence of cryoglobulinemia. Cryoglobulin tests are most often performed for many autoimmune and infection disorders rather than for neuropathic pain disorders. The requests for cryoglobulin tests are far less frequent than would be expected, which suggests that cryoglobulinemia in SFN could be an important but neglected clinical condition[18]. In this prospective observational study, we planned to investigate the distribution of the uncommon etiologiesof uncommon DSPSFN. The relationship between cryoglobulinemia and its clinical pain characteristics was also studied. We have also followed up on the clinical prognosis in these patients.

## Materials and methods

### Participants

Participants were prospectively recruited from consecutive patients referred from the outpatient clinic in the Department of Neurology at Chang Gung Memorial Hospital, Linkou between September 2012 and September 2014. The eligibility criteria included that the participant was between 18 to 70 years of age and presented with neuropathic pain for more than 1 month. The neuropathic pain was evaluated by questionnaire (described below). First, based on a history review or previous laboratory evidence, participants who had common etiologies of DSPSFNsuch as chronic diabetes mellitus or an impairment of glucose tolerance, alcoholism, malignancy, chronic renal diseases, dysthyroidism, connective tissue diseas e, vitamin B12 deficiency, paraproteinemia, hepatitis B or C virus (HBV, HCV) infection, HIV infection, neurotoxic drug exposure and erythromelalgia were excluded. The definition of uncommon DSPSFN was based on 1) the presence of symptoms and/or clinical signs of small-fiber damage (such as neuropathic pain, autonomic dysfunction, loss of pinprick sensation, thermal sensory loss, allodynia, or hyperalgesia); 2) the absence of large-fiber involvement (such as muscle weakness, loss of light touch and/or proprioceptive or vibratory sensation, hypoflexia, or areflexia)[2]. In addition, the conduction velocity in the large-diameter fibers, assessed by an NCS, should be normal.

**Fig 1:**
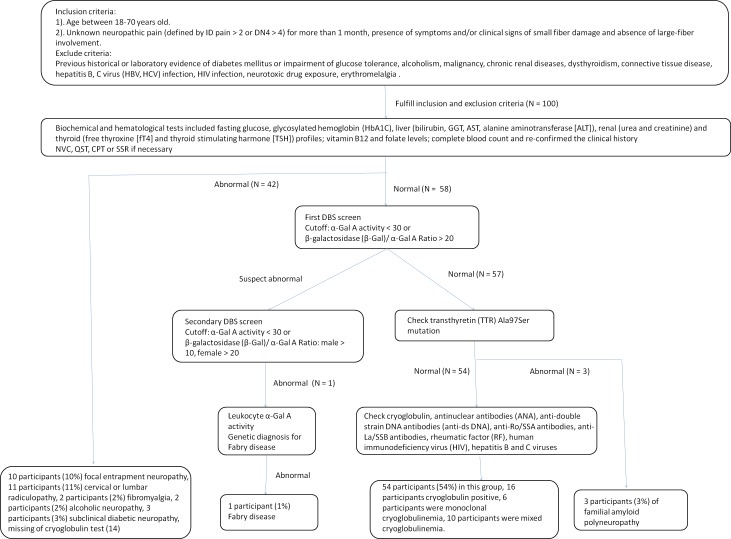
Study protocol for uncommon distal symmetric painful small-fiber neuropathy (DSPSFN).

Participants that fulfilled the inclusion criteria then received detailed biochemical and hematological tests including fasting glucose, glycosylated hemoglobin (HbA1C), liver (bilirubin, GGT, AST, alanine aminotransferase [ALT]), renal (urea and creatinine) and thyroid (free thyroxine [fT4] and thyroid stimulating hormone [TSH]) profiles; vitamin B12 and folate levels; and a complete blood count, and the clinical history was re-confirmed. We performed an HbA1c study for all participants, with 5.7–6.5% as the range used to identify patients with impaired glucose tolerance. Participants who had any abnormal findings in the above tests were also excluded. Then, a genetic screen for familial amyloid polyneuropathy and enzyme activity studies for Fabry disease were performed for participants who fulfilled the inclusion and exclusion criteria (described in Genetic investigations). After excluding the possibility of Fabry disease and familial amyloid polyneuropathy, participants received immunological tests for serum protein electrophoresis and a cryoglobulin test, as well as antinuclear antibodies (ANA), anti-double stranded DNA antibodies (anti-ds DNA), anti-Ro/SSA antibodies, anti-La/SSB antibodies, rheumatic factor (RF), human immunodeficiency virus (HIV), hepatitis B and C viruses. The study protocol and the number of participants are described in Fig 1. All of the results were categorized as normal or abnormal for statistical analysis. The duration of the disease follow-up was measured from the first time of visit to the time of the last visit. The activity of daily living (ADL) function was recorded from the patient’s description. The protocol for this study was approved by the Institutional Review Board of Chang Gung Memorial Hospital and University (License no. 100-4470A3 and 104-2462A3). Informed consent was obtained from all patients.

### Assessment questionnaire

Three pain-related questionnaires were applied in the current study. 1) The ID Pain questionnaire: The ID Pain questionnaire consists of 5 sensory descriptor items and 1 item relating to whether the pain is located in the joints (used to identify nociceptive pain). The tool was designed to screen for the likely presence of a neuropathic component to the patient’s pain[19]. 2) DN4 questionnaire: The DN4 is a self-report questionnaire. It consists of 7 items related to symptoms and 3 related to clinical examination. The DN4 questionnaire consists of 10 items and was easy to score for identifying neuropathic pain[20]. To differentiate neuropathic pain from other pain, we applied a cut-off value for the ID pain questionnaire of >2 points or > 4 points for the DN4 questionnaire. Both scales have been validated for the diagnosis of neuropathic pain with the ID pain questionnaire having 78% sensitivity and 74% specificity and the DN4 questionnaire 82–83% sensitivity and 81–90% specificity at the corresponding cut-off points[19–21]. 3) VAS scale: the pain visual analog scale is a unidimensional measure of pain intensity. It is a sensitive tool to measure various types of pain[22].

### Electrophysiological evaluation

A NCS was carried out on each subject’s left side using standard techniques [23]. Any value falling outside of the reference range (mean ± 2SD) in healthy individuals was considered abnormal. Those participants who had neuropathic pain confirmed by the ID pain and/or DN4 questionnaires but had a normal NCS were diagnosed with SFN. A quantitative thermal sensory test (QST) and current perception threshold testing (CPT) were selectively performed to measure the threshold temperatures for the sensations of warmth, cold, heat pain and cold pain [24, 25]. An autonomic function test including an RR interval variation (RRIV) and sympathetic skin responses (SSRs) were performed when appropriate. All results were recorded as normal or abnormal.

### Genetic investigations

Additional investigations were performed to determine the possible etiology of DSPSFN for all participants that fulfilled the inclusion and exclusion criteria. In particular, participants were screened for the Ala97Ser mutation, the most common cause of dominantly inherited transthyretin (TTR) mutations for familial amyloid polyneuropathy [26]. To study Fabry disease, a dry blood spot test to screen for total α-Gal A activity and β-galactosidase (β-Gal) activity was used, and a genetic study for the α-galactosidase A gene (GLA) exons including adjacent intronic regions was performed to confirm the Fabry disease diagnosis[27, 28]. The intentions of the study were explained to all participants, and informed consent was obtained from all participants before their inclusion in the study.

### Statistical analysis

All statistical analyses were performed using SPSS software (version 21.0). The continuous variables were expressed as the mean and standard deviation (SD). The categorical variables were presented by the numbers. Group comparisons of independent two-sample *t*-tests or Mann-Whitney *U*-test were conducted to compare the age, disease duration and pain score (ID pain, DN4 and VAS) between the cryoglobulinemia positive and negative group. A chi-square test was used to examine the gender distribution, autoantibodies results, and electrophysiological results between groups.

## Results

In total, 100 participants (65 males and 35 females, mean age: 50.3 ± 14.6 years old) were included and were determined to have uncommon DSPSFN with either an ID pain questionnaire score of >2 points or DN4 questionnaire score > 4 points for more than 1 month. Based on our study protocol, 46 participants were excluded due to a missing cryoglobulin test (n = 14) or a diagnosis of a focal entrapment neuropathy such as carpal tunnel syndrome, tardy ulnar palsy or tardy tarsal syndrome (n = 10), cervical or lumbar radiculopathy (n = 11), fibromyalgia (n = 2), alcoholic neuropathy (n = 2), subclinical diabetic neuropathy (n = 3), Fabry disease (n = 1), or familial amyloid polyneuropathy(n = 3). Finally, 54 participants were enrolled for analysis (37 males and 17 females, mean age: 53.2 ± 15.9 years old). The cryoglobulin test was positive in 16 participants (29.6%). In these participants, 6 participants had monoclonal cryoglobulinemia (Immunoglobulin M, IgM), and 10 participants had mixed cryoglobulinemia (IgM, IgA, IgG). We then divided the total participants into a cryoglobulinemia-positive and negative groupand compared their clinical characteristics. However, no significant age or gender differences were found between the two groups. In terms of the follow-up duration, the cryoglobulinemia-positive group had a shorter follow-up duration than the negative group (cryoglobulinemia-negative group: positive group = 4.4 ± 3.3 *vs*. 14.1 ± 2.2 months, p = 0.02). The ADL function in both groups, as described by the participants themselves, was normal until the last visiting date. Neither muscle weakness nor atrophy was detected by the clinicians. For the pain characterization, neither the ID pain nor the VAS scores was significantly different between the two groups, but the DN4 score was higher in the cryoglobulinemia positive group (p = 0.04) (Table 1).

**Table 1:** Demographics of the cryoglobulinemia-positive and -negative groups.

	Cryoglobulinemia-positive group (n = 16)	Cryoglobulinemia-negative group (n = 38)	P-value
Age	53.4 ± 4.0	53.1 ± 2.6	0.95
Gender (M:F)	10:6	27:11	0.54
Disease duration (month)	4.4 ± 3.3	14.1 ± 2.2	0.02*
ID pain	2.3 ± 1.3	2.4 ± 1.5	0.68
DN4	3.3 ± 2.3	3.1 ± 1.9	0.04*
VAS	3.5 ± 1.7	5.5 ± 2.3	0.77

*: p < 0.05

In the autoantibodies studies, 59% of the participants had abnormal anti-Ro/SSA and/or anti-La/SSB antibodies, but none fulfilled the criteria for Sjogren’s syndrome. The rate of abnormal RF was 3%. No significant difference was found between the two groups in ANA, anti-ds DNA, anti-Ro/SSA, anti-La/SSB autoantibodies or RF. In the cryoglobulinemia-positive group, one participant was positive for the HBV marker, and another participant was positive for the HCV marker. Both participants had the mixed type of cryoglobulinemia. In the electrophysiological studies, there was no significant difference in the QST or CPT between the two groups (p = 0.80 and 0.65, respectively) (Table 2). We then divided the participants based on the QST and CPT results and analyzed the neuropathic pain scores and autoantibodies. There was no significant difference between QST-normal and abnormal participants in pain scores or autoantibodies. In the CPT study, the DN4 questionnaire score was higher in the abnormal CPT group than the normal group by Mann-Whitney *U*-test (p = 0.02). There was no significant difference in the ID pain scores, VAS scores or autoantibodies results. In the enzyme activity study, we found a 57-year-old male patient with abnormal total α-Gal A activity and β-galactosidase (β-Gal) activity. The genetic analysis confirmed the diagnosis of Fabry disease. Three patients with the Ala97Ser mutation were also found using our study protocol, and the diagnosis of familial amyloid polyneuropathy was made.

**Table 2:** Results of autoantibodies and electrophysiological studies in the cryoglobulinemia-positive and -negative groups.

Study	Total cases	Cryoglobulinemia-positive group(N = 16)	Cryoglobulinemia-negative group(N = 38)	P-value*
ANA (N:A)	36:1	10:1	26:0	0.11
Anti-DS DNA (N:A)	13:0	6:0	7:0	1
Anti-SSA/SSB (N:A)	7:10	1:2	6:8	0.76
RF (N:A)	34:1	9:0	25:1	0.44
QST (N:A)	9:5	3:2	6:3	0.80
CPT (N:A)	8:12	2:2	6:10	0.65

ANA, antinuclear antibodies; Anti-DS DNA, anti-double stranded DNA antibodies; Anti-SSA, anti-Ro/SSA antibodies; Anti-SSB, anti-La/SSB antibodies; RF, rheumatic factor (RF); NCS, nerve conduction study; QST, quantitative thermal sensory test; CPT,current perception threshold testing;N, normal; A, abnormal.

* Chi-square test for cryoglobulinemia-positive and -negative group.

## Discussion

In this study, we investigated the clinical characteristics of cryoglobulin-related painful SFN. Using our study protocol, we found 3 participants with painful diabetic neuropathy and 2 participants with alcoholic neuropathy even though we had excluded those with diabetic mellitus and alcoholism through a detailed history and chart review. Focal entrapment neuropathy, radiculopathy and fibromyalgia could be presented as a distal symmetric neuropathic pain pattern for more than one month. After excluding participants with the most common etiologies of DSPSFN, we still had 16 participants that tested positive in the cryoglobulin test. Our findings showed that cryoglobulinemia-positive and negative SFN participants did not show a significant difference in age, gender, autoantibodies profile or electrophysiological study results. Although the cryoglobulinemia-positive group had a shorter follow-up duration, no ADL function impairment was described in either group. The DN4 scale, as a measure of the neuropathic pain, showed a higher score in the cryoglobulinemia-positive group than the negative group, but the pain severity measured by the VAS score did not show a significant difference between groups. The ID pain score also did not show a significant difference between groups. Using our structuralized protocol, we found one participant (1%) that had Fabry disease and three participants that had familial amyloid polyneuropathy (dominantly inherited transthyretin (TTR) mutations) presenting as DSPSFN. This is the first study to report the detection ratios of Fabry disease and familial amyloid polyneuropathy in this particular group of uncommon DSPSFN participants.

### Prevalence of DSPSFN

The prevalence of DSPSFN, either idiopathic or associated with systemic diseases, is unknown, but a recently study showed 11.7 cases/100,000 inhabitants/year at a tertiary referral center[2, 5]. The most common etiology of SFN could be related to metabolic factors such as diabetes mellitus or impaired glucose tolerance[1]. Our study protocol excluded past history of diabetes mellitus, but we still found 3 cases of diabetes mellitus without fully developed retinopathy, neuropathy or nephropathy, which was in alignment with previous literature that showed that diabetes mellitus is one of the common etiologies for painful SFN[2]. Two cases of fibromyalgia were identified. The relationship between fibromyalgia and painful SFN is controversial; either an autoimmune mechanism or generalized pain triggered by SFN have been proposed [29]. After excluding the possible etiological factors of SFN, our participants showed a relatively high positive ratio of anti-Ro/SSA and/or anti-La/SSB autoantibodies, which has not been previously reported. These participants could not fulfill the criteria for Sjogren’s syndrome because they lacked the clinical sicca syndrome. Until now, very few prospective studies evaluated the prevalence of the possible etiologies for SFN in a well-defined group of patients with painful SFN[30]. A similar study showed a specific protocol in painful SFN, which demonstrated that a structuralized algorithm could provide the ratios of different etiologies [29]. However, cryoglobulinemia could be one of the etiologies for painful SFN, but it was not studied in their protocol [2, 29]

### The clinical characteristics of cryoglobulinemia-positive DSPSFN

The ratio of positive cryoglobulin tests in DSPSFN is unknown. From our observational study, approximately 16% of patients had positive results, which indicated that the cryoglobulin test is an important but under-used test in determining the etiology of uncommon DSPSFN[18, 31, 32]. The characteristics of neuropathic pain in the cryoglobulinemia-positive group were not significantly different from that in the negative group. Only the DN4 pain questionnaire showed a significantly higher score in the cryoglobulinemia-positive group than the negative group. From the CPT study, the DN4 pain questionnaire score was higher in the abnormal CPT group, which suggested that the DN4 pain questionnaire could be more correlated with abnormal electrophysiological results and cryoglobulin test results. However, the VAS scores were not significantly different between the abnormal and normal CPT groups, which represented a discrepancy between the subjective pain severity and the electrophysiological results. This neuropathic pain could be successfully treated by anti-depressant or anti-epileptic medication. The long-term prognosis is quite good in our participants.

From our study, one patient with Fabry disease (1%) was found after excluding those with common causes of DSPSFN such as diabetes mellitus or immune-mediated or alcohol, drug and infectious-related painful neuropathy. The diagnosis of Fabry disease was further confirmed with an enzyme activity study and genetic study. One study showed that the prevalence of Fabry disease in participants with unexplained left ventricular hypertrophic cardiomyopathy was 1% [33]. Another study showed that the prevalence of Fabry disease in dialysis patients with end-stage kidney disease was 0.02% [34]. These findings suggested that screening for Fabry disease under a specific criterion could largely enhance the positive ratio. Because Fabry disease is now a treatable disease, the early detection of Fabry disease based on an effective screening protocol is very important. Our structuralized protocol provides the clinician a method to detect Fabry disease in patients with distal symmetric painful SFN. Three cases of familial amyloid polyneuropathy (3%) were identified from our study protocol. In these patients, the neuropathic pain symptoms occurred in the early phase of the disease and included progressive tingling and/or occasional throbbing/shooting pain or paresthesia/dysesthesia in the feet.

### The mechanism of pain in cryoglobulinemia-positive DSPSFN

The mechanisms of cryoglobulinemia in DSPSFN are unknown. Cryoglobulinemia generally leads to a systemic inflammatory syndrome characterized by fatigue, arthralgia, purpura, neuropathy and glomerulonephritis. The disease mainly involves small to medium-sized blood vessels and causes vasculitis due to cryoglobulin-containing immune complexes. In our participants, no systemic vasculitis, skin purpura or nephropathy was found in the four limbs, but distal symmetric pain was found. This is consistent with the previous findings. as patients with mild cryoglobulinemic syndrome had more frequent small-fiber sensory neuropathy than other active syndromes such as systemic vasculitis, skin purpura or nephropathy[32]. Three types of cryoglobulins have been found from clinical evaluations and could be classified based on the type of immunoglobulin in the cryoprecipitate[35]. Some authors have recommended classifying cryoglobulins into ‘simple monoclonal cryoglobulins’ (Type I) and ‘mixed cryoglobulins’ (Type II and Type III)[36]. In our study, most of the cryoglobulinemia belonged to “mixed cryoglobulins.” Cryoglobulins could be deposited in the endoneurial space. From a nerve biopsy study in patients with monoclonal dysglobulinemia and polyneuropathy, cryoglobulins were deposited in the endoneurial space as digitiform or granular structures observed under electron microscopic examination[37]. The deposition may compress the endoneurial vessels with a large amount of protein, leading to ischemic damage[37, 38]. Another possibility could be the interference with local microcirculation, which leads to ischemic injury to the peripheral nerves[37]. Other contributing factors such as increased local proinflammatory cytokine gene expression and a reduction in the intraepidermal nerve fiber density in SFN may be related to cryoglobulinemia-associated painful SFN[39]. Treatment of cryoglobulinemia-related painful SFN has been symptomatic amelioration using anti-depressant and anti-epileptic medication for the control of neuropathic pain. Several procedures for cryoglobulinemia removal or decreased production can be performed by plasmapheresis or steroid or cytotoxic medications, but the outcome is still undetermined[17].

The present study has several limitations. First, we were highly selective in our inclusion of a group with uncommon DSPSFN, excluding most common etiologies, which may bias the incidence of cryoglobulinemia in these patients. However, our intention in this observational study was to investigate the rare etiologies of DSPSFN. The incidence of Fabry disease is 1:40,000 to 1:117,000[40, 41]. Based on our protocol, we could enhance the detection ratios of Fabry disease (1%) and familial amyloid polyneuropathy (3%) in our participants. Second, we did not perform a skin biopsy or detailed molecular studies regarding channelopathies in our participants. We acknowledge that skin biopsy is the gold standard for the diagnosis of small-fiber neuropathy; however, it is also an invasive procedure that was not feasible for a clinical observational study [42]. Moreover, the sensitivity and specificity by skin biopsy for the diagnosis of small-fiber neuropathy is only 0.77 and 0.79,respectively[43]. A recent study has shown the linkage between multiple pain syndromes and channelopathies, such as those involving the voltage-gated sodium channel Na_v_1.7, which is preferentially expressed within the DRG and sympathetic ganglion neurons, in a substantial fraction of patients with idiopathic SFN[16]. We acknowledge these limitations, and these could be of interest in a future study. Third, although we followed up with our participants from 1 to 49 months (mean duration 11 months), the neuropathic pain symptoms in our participants could be a precursor to large fiber neuropathy such as chronic idiopathic axonal polyneuropathy. Sensory symptoms are more prominent in these patients, and in approximately 10% of these patients presenting with pain, neuropathic pain symptoms in particular occur earlier in the course of the disease [44]. Fourth, the number of participants in our study was not large enough to generalize these findings to clinical practice. This is a single hospital-based observational study, and the source of uncommon DSPSFN could be biased by the characteristics of a tertiary referral medical center. However, our study provided the proportional number of rare etiologies in a well-defined group of DSPSFN participants that excluded those with the most common etiologies. Our findings also established a baseline reference to help future researchers estimate the prevalence of Fabry disease, familial amyloid neuropathy and cryoglobulinemia in this group.

## Conclusions

Cryoglobulinemia is not a rare etiology determined by a well-defined structuralized protocol in uncommon DSPSFN. None of the participants with uncommon DSPSFN showed any sign of progression of muscle weakness and/or atrophy, gait disturbance, or autonomic dysfunction during the follow-up period. The long-term prognosis was fairly good. A detailed clinical history combined with molecular and genetic studies could help to determine the rare etiologies of uncommon DSPSFN.

## Supporting information

S1 Data (XLS)
